# Urothelial bladder carcinoma in childhood: a case report

**DOI:** 10.11604/pamj.2020.36.91.20416

**Published:** 2020-06-15

**Authors:** Aref Zribi, Sonia Ben Nasr, Issam Msakni, Sarra Karrit, Faten Gargouri, Sana Fendri, Mehdi Balti, Abderrazek Haddaoui

**Affiliations:** 1 Université de Tunis El Manar, Faculté de Médecine de Tunis, 1007, Tunis, Tunisie,; 2 The military hospital of Tunis, Department of medical oncology, Montfleury 1008, Tunisia,; 3 The military hospital of Tunis, Department of pathology, Montfleury 1008, Tunisia

**Keywords:** Pediatric, bladder, carcinoma, urothelial, surgery, prognosis

## Abstract

We report an exceptional case of transitional cell carcinoma of the bladder in a 14-years old boy without personal nor family history who consulted for a total hematuria. Work-up showed a bladder lesion sized 5cm with histology of urothelial cancer. Treatment consisted of a transurethral surgery with carcinologic complete resection. Patient is alive, free of disease with a follow-up of 36 months.

## Introduction

Bladder cancer (BC) had usually an urothelial lineage and affects adults in 5^th^-6^th^ decade which is representing the 9^th^ most common malignancy worldwide [1]. Occurrence of urothelial cancers is exceptional in younger patients, below 20 years [2]. We report a new observation in a young patient, 14 years-old.

## Patient and observation

**Figure 1:**
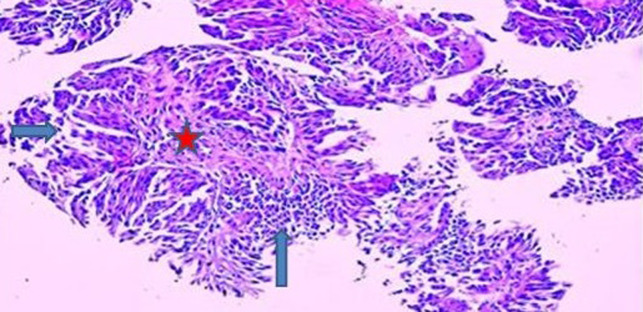
superficial papillary urothelial tumor (pTa) (Hex100) (papillary axis, superficial urothelial tumor)

**Figure 2:**
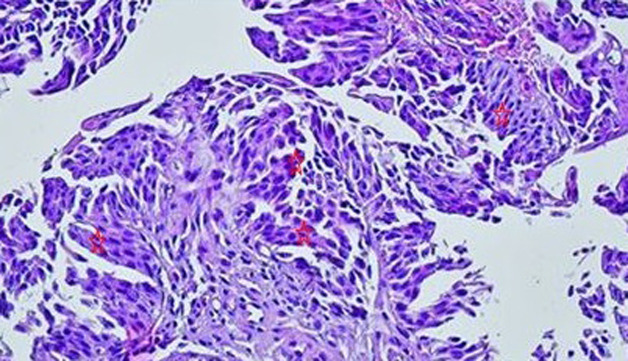
the nuclei of urothelial cells are globally monomorphic and non-mitotic (low grade) (Hex250)

A 14-year-old boy, consulted for a persistent and painful total hematuria 3 months before consultation. There was no personal nor family history of benign or malignant disease, nor an exposition to passive smoking, chemotherapy, radiation or chemical toxics. Physical examination revealed a normal abdominal and genitourinary examination. Abdominal ultrasonography showed a 12x11x7 mm sized bladder mass beside the left urethral orifice without vascularization. The results of a complete blood count, liver function, and renal function tests were within normal limits. The urine test revealed only hematuria, and urine cytology showed no evidence of malignancy. A computed tomography scan revealed a single bladder mass of 10 mm without enlargement of the lymph nodes or metastasis. The biopsy concluded to an urothelial carcinoma grade II pTa without stromal invasion. Transurethral resection of the bladder mass was performed. The tumor was characterized by uniformly enlarged nuclei with moderate differences in shape, and chromatin distribution. The tumor didn't invade sub-epithelial connective tissue and the muscle was not removed. The histopathology concluded to a non-invasive (pTa), low grade (GII), papillary urothelial carcinoma (Figure 1, Figure 2). No adjuvant treatment was needed. The child is currently asymptomatic and followed-up with ultrasonography and cystoscopy. Patient is alive free of disease with a follow-up of 36 months.

## Discussion

Transitional cell carcinoma of the bladder in the first 2 decades of life is distinctly rare and not well characterized. Tumor incidence is 0.4% in individuals younger than 20 years and 0.03% in those younger than 16 years [2]. This tumor was only described in case reports and small series. They are typically characterized by low-grade histology and cure with complete surgical resection. No predisposing factors are known for children. Adult risk factors such as smoking, radiation, and chemical and occupational exposures are not typically involved in pediatric BC. Association with rare genetic syndromes like Costello or Hinman syndrome, and Cowden disease were reported. [3]. Li Fraumeni syndrome, Fanconi anemia, and hereditary non-polyposis colorectal carcinoma syndrome were associated with pediatric BC. These syndromes could be diagnosed with genetic testing for p53 mutations, functional (DEB) testing, and analysis of tumor microsatellite instability (MLH1, MSH2, MSH6, PMS2), respectively [4]. The most common symptom is a gross haematuria usually not associated with dysuria [5]. Tumor is most often located in the trigone (75%) [6]. A delay in the diagnosis is common in paediatric population probably because of urinary tract infection (15%) and microscopic haematuria (5%) [7]. Bladder ultrasound combined with cystoscopy identify nearly all primary lesions.

Definitive diagnosis is performed by cystoscopy which allows evaluation of tumor extension, excision and biopsy [5]. Urine cytology is not a useful diagnosis tool in young patient because of the predominance of low grade lesions [6]. According to the 1973 World Health Organization (WHO) classification, pediatric BC was histologically graded as well differentiated (grade 1), moderately differentiated (grade 2), and poorly differentiated (grade 3). However, this classification has been updated by WHO/International Society of Urologic Pathologists consensus classification published in 2004 to papillary urothelial neoplasms of low malignant potential, low-grade papillary urothelial carcinomas, and high-grade papillary urothelial carcinomas [8]. Papillary urothelial neoplasms of low malignant potential are frequent particularly in teenagers. These tumors have a low rate of progression to higher grade and stage and thus differs from papillary urothelial carcinoma [6]. Alterations of INK4, p21 and p27 genes are associated with an increased risk of reocurrence [5, 6, 9]. A higher ki67 expression level in elderly than in young patients was reported and could explain the better outcome in pediatric population [9]. The main treatment modality is transurethral resection (TUR) of tumor because of low grade of malignancy and low recurrence rate [10]. Because of limited data, no standards exist for the use of adjuvant medical therapy or intravesical immunotherapy. Radical cystectomy, partial cystectomy, and chemotherapy may be reserved as treatment options for children with high-grade or muscle-invasive carcinomas [1, 8]. Close follow-up is necessary without consensus on follow-up methods in pediatric patients owing to the low incidence in the literature [1]. Recurrence rate of 2.6% to 13% were reported in patients aged less than 20 years with epithelial tumors versus 40% to 70% in adults [8]. Ultrasonography (USG) is the most commonly used modality for the postoperative follow-up of pediatric patients because of its noninvasiveness and high sensitivity. Computed tomography is not recommended for the follow-up because of the risk of exposure to ionizing radiation and high cost involved without gain in sensitivity when compared to USG. Urinary cytological screening is often not helpful because of the low-grade of tumors, and a low sensitivity ranging between 6% and 38%. Cystoscopy remains the gold standard for the postoperative follow-up, despite its disadvantages including requirement for general anesthesia and possibility of developing urethral trauma.

In practice, cystoscopy may be done if recurrence in suspected on bladder ultrasound [3, 7, 8, 11]. Ander *et al*. [12] proposed similar follow-up for pediatric and adult patients. They monitored pediatric low-grade BC with USG and cystoscopy at 3 and 9 months, followed by USG twice a year and cystoscopy once a year for the subsequent years. For high-grade tumors, they preferred doing USG and cystoscopy at 3, 6, and 12 months postoperatively and cystoscopy twice a year in the following years [8]. High-grade bladder carcinomas can be lethal and require more aggressive treatment and monitoring when compared to low-grade carcinomas [4]. High ki67 expression and low cycline D1 were associated with a greater risk of recurrence in young patients [10]. By searching in the recent literature of pediatric BC cases, 82 cases were found (Table 1). To the best of our knowledge, our case is the first published BC in childhood. Our case was similar to cases reported in the literature treated with transurethral resection without adjuvant treatment. The patient is currently asymptomatic and followed-up with ultrasonography and cystoscopy. Overall survival was three years without evidence of recurrence. Survival rate is above 95% at 5 years [1].

**Table 1:** previously published case series of pediatric urothelial carcinoma

Ref	Cases/sex	Age (years)	Diagnostic method	Grading	Treatment	Outcome (follow up years)
Hoenig DM (7)	5/M	11-18	USG/CT	G1-G2	TUR	4NR(6Y)/1R(3Y)
Fine SW (9)	23/19M4F	4-20	CYS	20G1-3G3	TUR	3R (1-7y)
Lerena J (5)	6/4M2F	6-17	US/CYS	G1	TUR	NR
Park S (6)	2/M	13-16	US/CT/CYS	G1	TUR	NR(1Y)
Rifat UN (10)	2/M	5-12	US/CYS/MRI	G1	TUR	1R/NR (3Y)
Aguiar L (3)	1/F	3	MRI/CYS	G3	TUR	NR (1Y)
Polat H (11)	9/5M4F	12-17	US	Low grade	TUR	NR (7y)
Uçar M (8)	4/2M2F	10-17	US	G1	TUR	NR (15Y)

USG: ultrasonography; CT:CT scann; CYS: cystoscopy; MRI: magnetic resonance imaging; TUR: transurethral resection; NR: no recurrence; R: recurrence

## Conclusion

Although bladder tumors are rare in children and adolescents, it should be considered in the case of painless gross hematuria. Pediatric BC have a good prognosis owing to the low malignancy grade and the low rate of recurrence. USG followed by cystoscopy are the ideal diagnostic tools. Endoscopic resection is the standard of treatment. Follow up must be clinical with periodic evaluation. Periodic cystoscopy is indicated only in cases of clinical or ultrasonographic suspicion of recurrence.
